# Aqua-MC as a simple open access code for uncountable runs of AquaCrop

**DOI:** 10.1038/s41598-025-08995-z

**Published:** 2025-07-10

**Authors:** Vahid Adabi, Hadi Ramezani Etedali, Asghar Azizian, Faraz Gorginpaveh, Ali Salem, Ahmed Elbeltagi

**Affiliations:** 1 Department of Water Sciences and Engineering, Imam Khomeini International University, Qazvin, Iran; 2 Department of Civil, Construction and Environmental Engineering, Center for Complex Hydrosystems Research, University of Alabama, Tuscaloosa, USA; 3 Civil Engineering Department, Faculty of Engineering, Minia University, Minia, 61111 Egypt; 4 Structural Diagnostics and Analysis Research Group, Faculty of Engineering and Information Technology, University of Pécs, Pecs, Hungary; 5 Agricultural Engineering Department, Faculty of Agriculture, Mansoura University, Mansoura, 35516 Egypt

**Keywords:** Crop models, Monte-Carlo, Uncertainty, Sensitivity analysis, Multi-run, Computer science, Software

## Abstract

Understanding uncertainty in crop modeling is essential for improving prediction accuracy and decision-making in agricultural management. Monte Carlo simulations are widely used for uncertainty and sensitivity analysis, but their application to closed-source models like AquaCrop presents significant challenges due to the lack of direct access to source code. This study introduces Aqua-MC, an automated framework designed to facilitate Monte Carlo simulations in AquaCrop by integrating probabilistic parameter selection, iterative execution, and uncertainty quantification within a structured workflow. To demonstrate its effectiveness, Aqua-MC was applied to wheat yield modeling in Qazvin, Iran, where parameter uncertainty was assessed using 3000 Monte Carlo simulations. The DYNIA (Dynamic Identifiability Analysis) method was employed to evaluate the time-dependent sensitivity of 47 model parameters, providing insights into the temporal evolution of parameter influence. The results revealed that soil evaporation and yield predictions exhibited the highest uncertainty, while transpiration and biomass outputs were more stable. The study also highlighted that many parameters had low impact, suggesting that reducing the number of free parameters could enhance model efficiency. Despite its advantages, Aqua-MC has some limitations, including its computational intensity and reliance on the GLUE method, which may overestimate uncertainty bounds. To improve applicability, future research should focus on parallel computing, cloud-based execution, integration with machine learning techniques, and expanding Aqua-MC to multi-crop studies. By overcoming the limitations of closed-source models, Aqua-MC provides a scalable and efficient solution for performing large-scale uncertainty analysis in crop modeling.

**Supplementary Information:**

The online version contains supplementary material available at 10.1038/s41598-025-08995-z.

## Introduction

Modeling is the use of equations or sets of equations to represent the behavior of a system. The development of crop growth simulation models has been a natural progression of scientific research. Analyzing behavior of input parameters and output time series is an important purpose for better identification of model concepts, better calibration in different conditions, and optimized responses^1^. Uncertainty Analysis (UA) and sensitivity analysis (SA) methods are common approaches for reaching this purpose that are widely used for the assessment of complex environmental models^2–5^. Combining multiple SA methods, such as Morris or EFAST, can provide more comprehensive insights for model calibration^3^ and the time-series SA can the changes of parameter sensitivies during different times of year which could affect outputs^6^.

For applying UA and SA methods usually we need to run the model many times (maybe more than several thousand runs at least)^7^. A common approach for handling such extensive simulations is the Monte Carlo method, which relies on random sampling to explore the variability of model outputs based on uncertain input parameters. This method has been widely used in various studies to estimate probabilistic distributions of outcomes and assess the robustness of models or understand the uncertainties of a decision^8–11^. It is not out of access for open-source models by using the common concept of loop in programming. However, reaching too many runs in closed-source models is not easy because of not accessing their codes. Manually repeating tasks in closed-source models is time-intensive and prone to error^12^. In this study, we present a simple approach that is programmed in MATLAB software and assists AquaCrop users in applying Monte-Carlo-based methods or multi-run for better analyze of this model in desired conditions (e.g. crop, soil, climate, management). AquaCrop has undergone rigorous testing in research, demonstrating remarkable success, and was chosen as a validated model.

The importance of understanding the uncertainty of crop modeling has increased in the past decade^13^. Monod et al. (2006) provided various drivers of uncertainty that included crop models which were alternative model structures, the initial values and variables^14^. The concept of uncertainty has been used for modelers in various research that included various techniques, such as Bayesian techniques^15–17^. Sensitivity analysis and uncertain quantifies the impact on the results of the model that can involve soil^18^, management^19^, and initial values^20^.

AquaCrop^21^, specifically designed as a crop water productivity and yield response model, has undergone extensive evaluation, testing, and application in numerous research and management contexts, encompassing diverse environmental scenarios. It is also known as an effective model for water-limited environments^22^ and have been used for various crops including cabbange^23^, soybean^24^, wheat^25^ and maize^26,27^, and quinoa^28^.

AquaCrop, as one of the closed-source models, has several challenges for advanced modeling workflows. Challenges with modifying the codes directly, limiting automations and script flexibility has made this model hard to deal with. As a result, performing high-volume simulations requires manual repetition of individual runs, which is labor-intensive and prone to human error. This inefficiency limits scalability and makes comprehensive uncertainty and sensitivity analyses impractical for many researchers.

AquaCrop-OS^29^ is developed to compensate for FAO’s version limitations like lack of origin codes. Its authors in detail explained their reasons for providing the open-source version in their paper. Surely, a compiled code takes freedom of action from users in comparison with open access code. One of their limitations is to Apply multi-run, like many thousand random runs for Monte-Carlo methods. Multi-run simulations involve repeating models running with different inputs. And Monte-Carlo simulation focus on uncertainty quantification. Whereas many engineering and environmental software are closed source and they have not any accessible code for ordinary users^30^. Also, many users want to use only the main version of models for many reasons as the originality and validity of main models and the facilitation of utilizing their user interface.

A key limitation of traditional SA methods is that they often pass parameter influence at a single point in time. However, in dynamic systems, such as crop growth models, parameter importance can change significantly across different growth phases. To address this, Dynamic Identifiability Analysis (DYNIA) provides a time-resolved approach to SA and tracks how the influence of each parameter evolves over the entire simulation period. Instead of evaluating parameters based on a single aggregated output, DYNIA calculates sensitivity at each time step, which reveals parameters with the highest influential at different crop growth stages. This allows researchers to have a better understanding of the model behavior, refine calibration strategies, and improve forecasting accuracy by targeting the most relevant parameters at each stage^1,31^.

In this study, we ran the AquaCrop model for thousands of inputs with a provided code. The SA method was DYNIA which has been tested before in various studies. We answered this question of how users can utilize and multi-run the main version of AquaCrop with the least human and manual actions. We offer this simple code for the practical use of closed-source models for different purposes, from crop models to hydrological or even non-agricultural and non-environmental purposes.

## The AquaCrop model

AquaCrop distinguishes itself from other crop models through its exceptional and unique features. One notable advantage is the simulation of canopy expansion using proportional green canopy cover, in contrast to the traditional leaf area index. This method enables a direct link between the outputs of the simulated model and the data obtained from field observations and remote sensing. One important aspect is the use of vegetation indices, like NDVI, derived from satellite observations, which show a strong connection with the proportion of green canopy cover. Also proportional green canopy cover provides scalable alternative to leaf area index. This facilitates the quick calibration and validation of AquaCrop outputs across extensive regional areas^29,32–38^.

### AquaCrop under stress conditions

AquaCrop also surpasses other water-driven crop models in its comprehensive consideration of water stress impacts on transpiration^39^. It incorporates the effects of stomatal closure as well as factors such as reductions in leaf expansion and premature canopy senescence. Furthermore, AquaCrop accounts for the dynamic effects of various environmental stressors, with a particular focus on water and temperature, as well as the impact of elevated atmospheric carbon dioxide concentrations on crop water productivity^40–43^.

### Stress thresholds

The model indirectly addresses the effects of soil fertility and salinity stresses through local calibration to relative biomass under different conditions. AquaCrop simulates each environmental stress effect by specifying upper and lower thresholds, as well as a shape parameter that determines the magnitude of the stress effect on crop growth processes. These thresholds and responses are specific to each stressor, such as root zone soil moisture depletion for water stress and soil electrical conductivity for salinity stress. Importantly, AquaCrop recognizes that stress thresholds and responses vary among crop types, reflecting their varying sensitivities to different environmental stress effects^44,45^.

### Inputs

The input parameters in AquaCrop can be categorized into meteorological conditions, soil characteristics, crop-specific parameters, and management practices. Meteorological inputs (such as evapotranspiration, temperature, and rainfall) influence water balance and crop development. Soil parameters (e.g., water depletion factors, salinity thresholds, and anaerobiotic conditions) changes moisture availability and root function. Crop parameters (e.g., canopy cover, growth coefficients, harvest index, and water productivity) determine biomass accumulation and yield. Management practices (e.g., irrigation strategies, plant density, and fertilization) impact water stress levels and nutrient availability. These parameters collectively influence model outputs, including evapotranspiration, soil evaporation, biomass production, and grain yield, with sensitivity analysis identifying the most influential variable^46,47^.

### Aqua-MC

This study utilized the stand-alone version of AquaCrop, which is a closed-source model. Aqua-MC automates simulations without modifying the internal computational framework of AquaCrop, ensuring full compatibility with its standard outputs. The Aqua-MC package comprises a set of accessible codes that facilitate the application of methods of Monte Carlo or other objectives in multi-runs of AquaCrop simulations. This algorithm is made possible through four key steps:

1. Selecting desired input parameters for creating treatments.

(function Sampling_Treatments_AAT)

2. Copying empty model, import intended values and creating treatments.

(function Write_Treatments)

3. Run each treatment one by one.

(function Run_All_exe)

4. Obtain results, apply statistical, mathematical or graphical methods for concluding.

(function Output_Read_and_Conclusion)

**Fig. 1:**
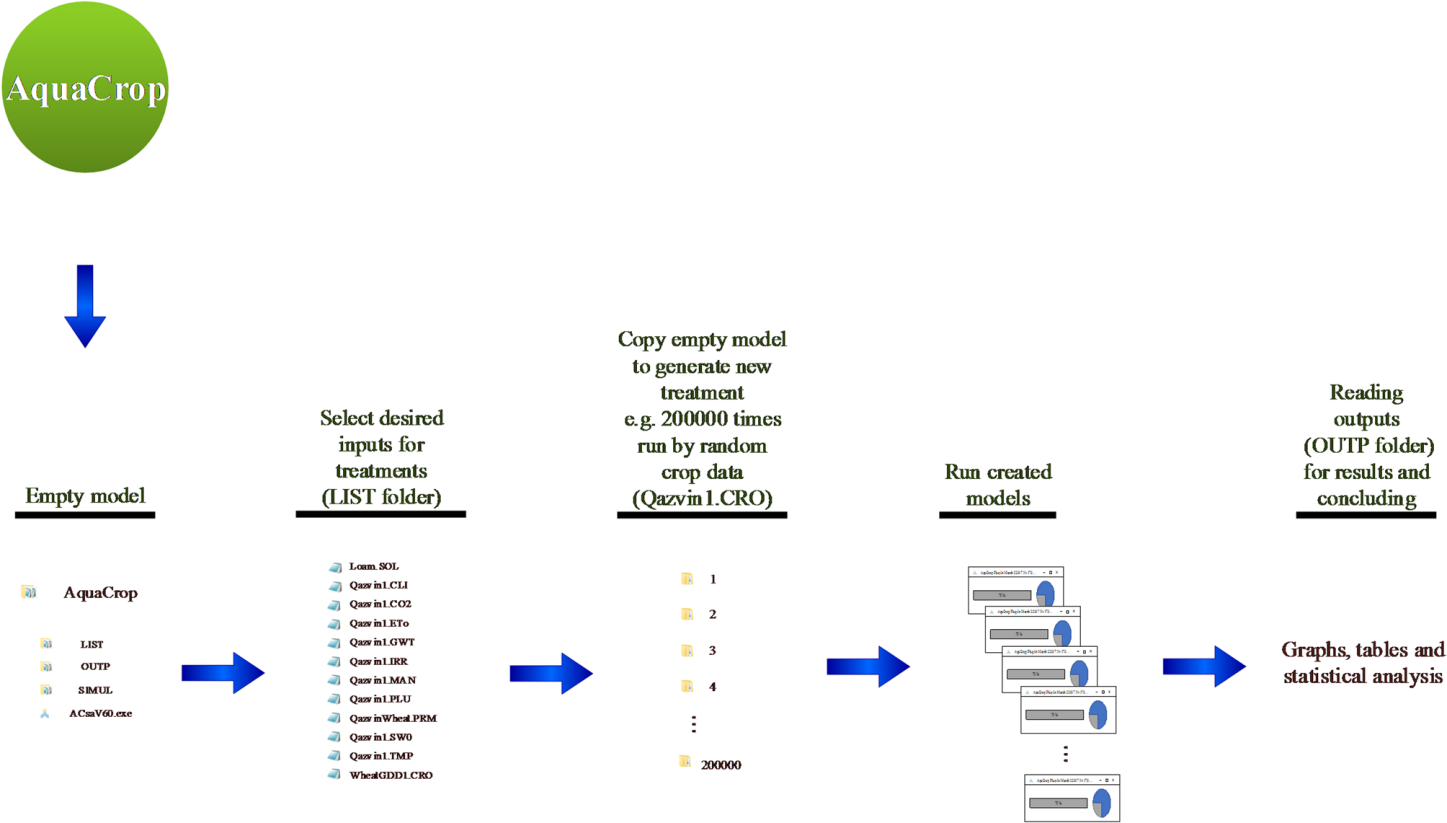
Diagram of the procedure for applying Monte-Carlo methods on AquaCrop. Based on this, Aqua-MC codes programmed by MATLAB software.

The key point to note is that AquaCrop can be executed without any manual intervention at any stage. The entire process, from creating a project file in AquaCrop to applying Aqua-MC, is automated through programming software like MATLAB. Figure 1 provides a visual representation of the process for better understanding.

Aqua-MC involves parameter sampling, treatment creation, automated execution, and result extraction and analysis. These steps are described as follows:

The first step involves selecting the desired input parameters to create treatments. For instance, if we want to run AquaCrop 200,000 times with random crop data, this function comprises three sections. Firstly, it identifies the range of parameters (prior distributions) and constant values. Secondly, it determines the sampling strategy for the parameters, and finally, it saves the sample matrix.

In the second step, we should copy the empty model, import the intended values, and create the treatments, so we can generate the necessary files for each treatment and prepare them for execution.

The “Run Each Treatment” function that enters the model includes the treatment and running one by one. We should provide precise information about the runtime for each treatment, but as we do not have access to the model’s source code, we need to define an approximate runtime. This approximate runtime can be 12 s for a default pause which alleviates the pressure on the CPU of the system.

Finally, we need to obtain the results and apply statistical, mathematical, or graphical methods to project the conclusions. This can involve reading the outputs from the executed treatments and analyzing them using appropriate techniques.

The automation process uses MATLAB to generate parameter sample matrix using Monte-Carolo or any other sampling techniques. Then users specify the range and distribution (such as uniform, normal, …) for each input parameter. And the MATLAB function creates a structured dataset of N parameter sets. The advangtages would be eliminating human error in parameter entry, allowing for complex probabilistic sampling instead of fixed manual values, and ensuring reproducibility with stored sampling scripts.

The Run Each Treatment function in this model is designed to execute multiple AquaCrop simulations while preventing overlapping runs sequentially. Before execution, the function generates a unique folder for each treatment that ensures the input file and output results are separate, then function runs treatments once at a time by invoking the AquaCrop’s executable through system commands in MATLAB while a loop structure monitors when each run is completed before launching the next. After that, the function checks for the completion status of each run before initiating the next simulation. A built-in waiting mechanism is now used to ensure each execution is fully completed before the next treatment begins. This sequential execution can be modified to use parallel computing.

DYNIA (Dynamic Identifiability Analysis) is a time-dependent sensitivity analysis method that evaluates how parameter influence evolves over time. First, N parameter sets are randomly sampled from the feasible parameter space and used in Monte Carlo simulations. The support set is determined based on an error metric (e.g., Mean Absolute Error or RMSE) over a moving time window (e.g., ± 10 time steps). The top-performing (best-fitting) parameter sets are selected, and their cumulative distribution function is computed to transform the raw parameter values into a probabilistic space.

Next, parameter values are divided into M containers across time, and the gradient of each parameter (Gi, m) is computed, revealing how its influence changes. The Identifiability Index (IDI) quantifies the degree of constraint on each parameter over time, with higher IDI values indicating greater influence. Finally, a time-parameter diagram is generated, showing how sensitivity fluctuates across simulation periods. This method helps identify critical growth phases, improve model calibration, and enhance uncertainty quantification by focusing on the most relevant parameters at each time step.

## Results and discussion

DYNIA is well-suited for studying wheat production in Qazvin due to the region’s seasonal climate variability and water-limited agricultural conditions. The crop is also sensitivie to time dependent factors such as rainfall distribution, irrigation scheduling, soil moisture fluctuations, and temperature extremes. DYNIA enables identification of most critical parameters at each time step, providing insights for targeted calibration and improving irrigation and management strategies.

Aqua-MC assessment needs to be utilized for executing sensitivity analysis methods on the grain yield of Qazvin wheat. The dynamic Identifiability Analysis (DYNIA) method includes an interest visualization for the sensitivity of parameters in each time step considered our choice. It was introduced to assess the dynamic variations in the identifiability of parameters as time progresses^48^.

The assessment of parameter identifiability involves calculating an objective function. One of the functions was mean absolute error but not for the entire calibration period, but for each time step. The resulting gradients are represented through varying shades of grey, where a higher gradient and darker shading indicate a higher degree of parameter identifiability. This process yields a time-parameter diagram that exhibits distinct patches with different shades of grey.

It is important to acknowledge that this method solely evaluates the global marginal distribution which is not considering the dependency of the parameter. A more comprehensive analysis of parameter dependence or interaction can be achieved by thoroughly investigating the response surface or the variance-covariance matrix. The movement of identifiability regions for different parameters in similar or opposite directions during specific periods may indicate parameter interaction.

Moreover, the DYNIA plot can be enhanced by incorporating 90% confidence limits. These limits exhibit narrower widths during periods of parameter identifiability and wider widths during periods where identification is challenging. To quantitatively assess these differences, a measure based on one minus the normalized distance between the confidence limits can be employed. A lower value of this measure, stemming from narrower confidence limits, indicates that the corresponding data period contains valuable information pertaining to the analyzed parameter. Consequently, these plots are referred to as information content plots in subsequent discussions.

The 90% confidence limit are computed to quantify the uncertainty in parameter sensitivity over time and limits the distribution of sensitivity gradeints across all Monte Carlo simulations at each time step. The calculation starts with large number of simulations with randomly sampeled arameter values, then at each time step, the gradients of parameter influence are calculated across all simulations. The 5th and 95th percentile of gradient values are extracted as lower and upper bounds of confidence interval. The mean sensitivity gradient is plotted as the central trend and the the shaded region shows the confidence level.

In this study, the DYNIA method was implemented using 3000 random runs to analyze the crop parameters of wheat in the Qazvin region. The SAFE (Sensitivity Analysis for Everybody) toolbox developed by Pianosi et al. (2015) was utilized for this purpose^49^. This toolbox serves as a valuable resource for implementing Monte-Carlo-based methods within the MATLAB software environment and is highly compatible with the results obtained from the preceding procedure.

**Fig. 2:**
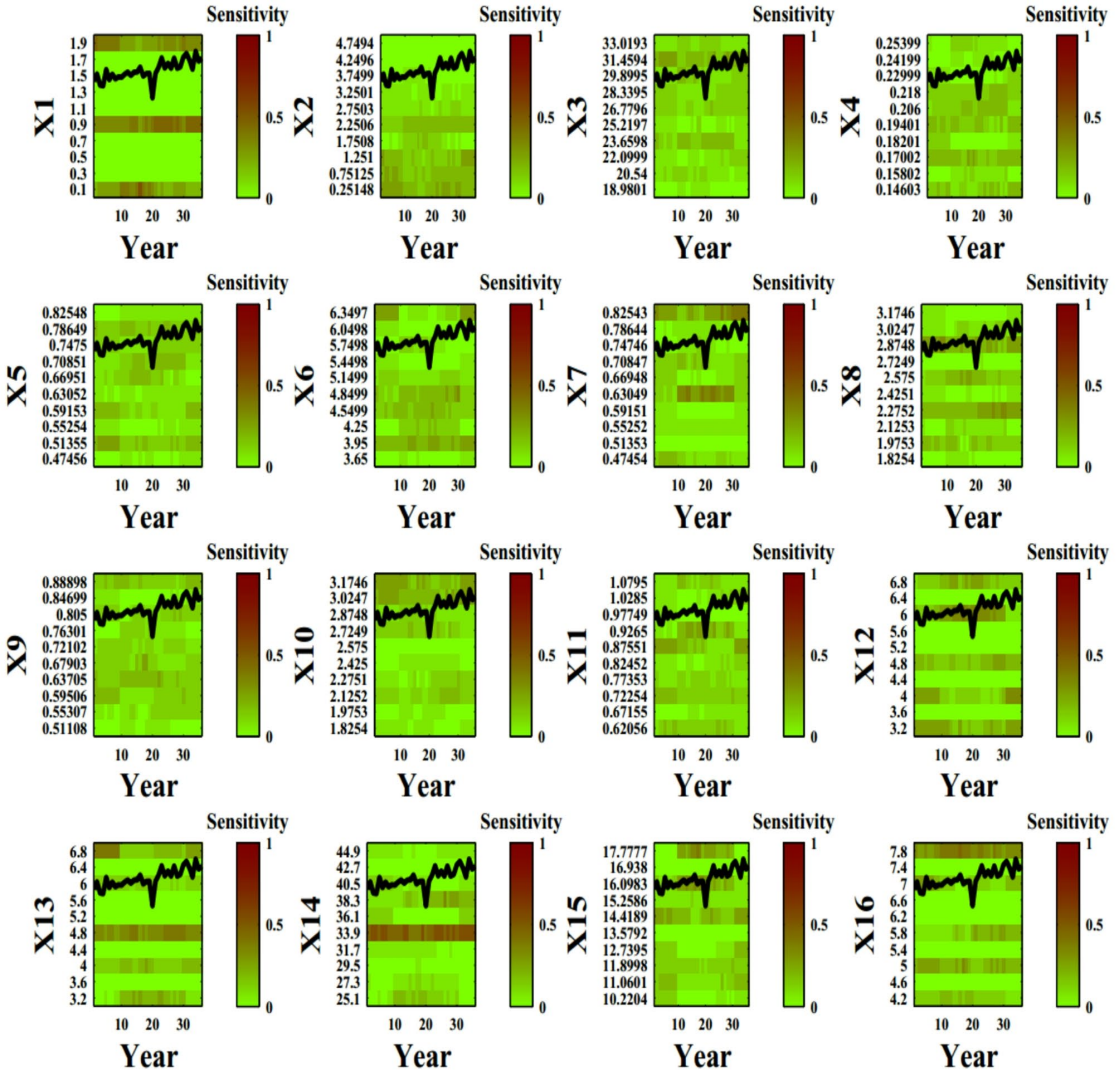

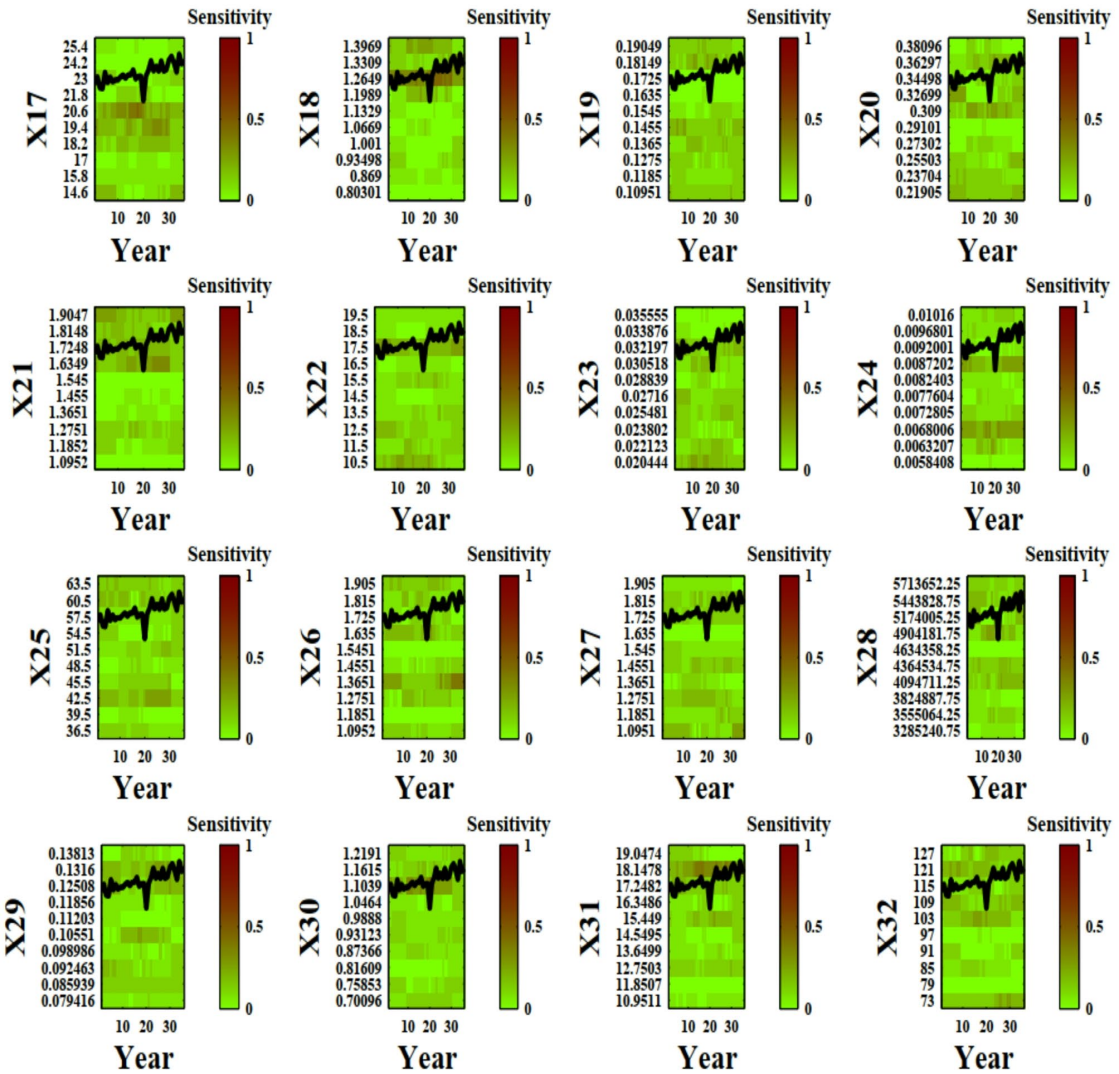

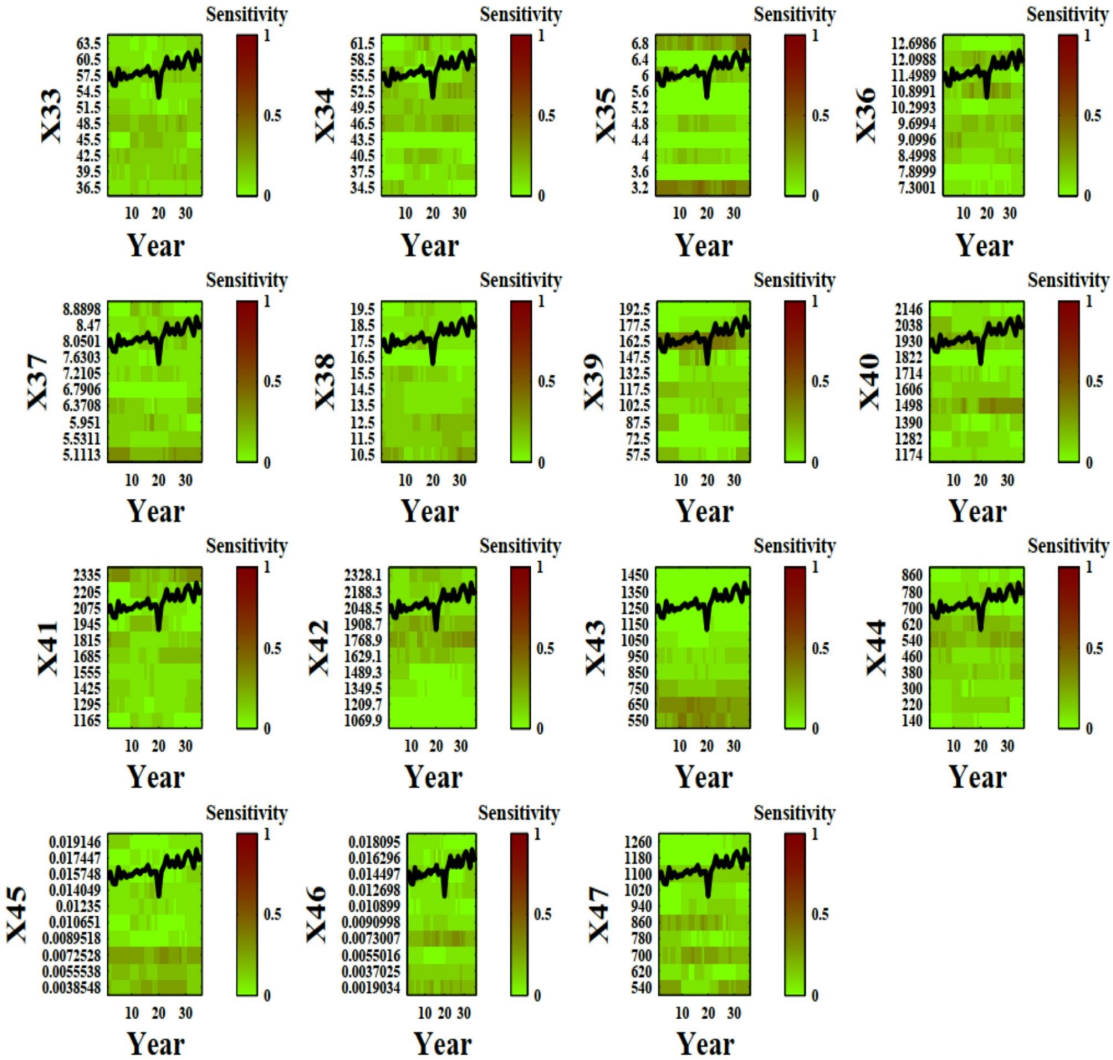
DYNIA plots for each input parameters. Vertical axis and horizontal axis display range of parameters and time period, respectively. From green to red, the sensitivity of parameters increases in the 36 years simulation of wheat yield in Qazvin (The names of parameters are in attachments).

Figure 2 visually depicts the sensitivity of the 16 input parameters at each value and time step, providing valuable insights into the dynamics of parameter influence in the system under investigation. Parameters of X1 to X47 have been introduced previously in the literature^2^ and are shown in Table 1. The selection of variables were chosen to be relevant for crop modeling of wheat in Qazvin to capture key aspects of crop growth, water dynamics, environmental interactions, and water stress. The calibration period was based on the need to capture both inter-annual climate variability and water availability trends in Qazvin. The duration was chosen to enhance the model’s ability to predict crop performance under future conditions.

**Table 1:** Name of crop parameters that able to vary in this study (* pararmeters must be integer number that aquacrop can’t accept float number for them).

	Name of crop parameters
X1 *	Soil water depletion factors (p) are adjusted by Eto
X2	Base temperature (°C) below which crop development does not progress
X3	Upper temperature (°C) above which crop development no longer increases with an increase in temperature
X4	Soil water depletion factor for canopy expansion (p-exp) - Upper threshold
X5	Soil water depletion factor for canopy expansion (p-exp) - Lower threshold
X6	Shape factor for water stress coefficient for canopy expansion (0.0 = straight line)
X7	Soil water depletion fraction for stomatal control (p - sto) - Upper threshold
X8	Shape factor for water stress coefficient for stomatal control (0.0 = straight line)
X9	Soil water depletion factor for canopy senescence (p - sen) - Upper threshold
X10	Shape factor for water stress coefficient for canopy senescence (0.0 = straight line)
X11	Soil water depletion factor for pollination (p - pol) - Upper threshold
X12 *	Vol% for Anaerobiotic point (* (SAT - [vol%]) at which deficient aeration occurs *)
X13 *	Minimum air temperature below which pollination starts to fail (cold stress) (°C)
X14 *	Maximum air temperature above which pollination starts to fail (heat stress) (°C)
X15 *	Minimum growing degrees required for full biomass production (°C - day)
X16 *	Electrical Conductivity of soil saturation extract at which crop starts to be affected by soil salinity (dS/m)
X17 *	Electrical Conductivity of soil saturation extract at which crop can no longer grow (dS/m)
X18	Crop coefficient when canopy is complete but prior to senescence (KcTr, x)
X19	Decline of crop coefficient (%/day) as a result of ageing, nitrogen deficiency, etc.
X20	Minimum effective rooting depth (m)
X21	Maximum effective rooting depth (m)
X22 *	Shape factor describing root zone expansion
X23	Maximum root water extraction (m3water/m3soil.day) in top quarter of root zone
X24	Maximum root water extraction (m3water/m3soil.day) in bottom quarter of root zone
X25 *	Effect of canopy cover in reducing soil evaporation in late season stage
X26	Soil surface covered by an individual seedling at 90% emergence (cm2)
X27	Canopy size of individual plant (re-growth) at 1 st day (cm2)
X28 *	Number of plants per hectare
X29	Canopy growth coefficient (CGC): Increase in canopy cover (fraction soil cover per day)
X30	Maximum canopy cover (CCx) in fraction soil cover
X31	Water Productivity normalized for ETo and CO2 (WP*) (gram/m2)
X32 *	Water Productivity normalized for ETo and CO2 during yield formation (as % WP*)
X33 *	Crop performance under elevated atmospheric CO2 concentration (%)
X34 *	Reference Harvest Index (HIo) (%)
X35 *	Possible increase (%) of HI due to water stress before flowering
X36	Coefficient describing positive impact on HI of restricted vegetative growth during yield formation
X37	Coefficient describing negative impact on HI of stomatal closure during yield formation
X38 *	Allowable maximum increase (%) of specified HI
X39 *	GDDays: from sowing to emergence
X40 *	GDDays: from sowing to maximum rooting depth
X41 *	GDDays: from sowing to start senescence
X42 *	GDDays: from sowing to maturity (length of crop cycle)
X43 *	GDDays: from sowing to flowering
X44 *	Length of the flowering stage (growing degree days)
X45	CGC for GGDays: Increase in canopy cover (in fraction soil cover per growing-degree day)
X46	CDC for GGDays: Decrease in canopy cover (in fraction per growing-degree day)
X47 *	GDDays: building-up of Harvest Index during yield formation

Figure 2 illustrates the time-dependent sensitivity of 47 input parameters in the AquaCrop model for wheat in Qazvin over a 36-year simulation period. The vertical axis represents different input parameters, while the horizontal axis corresponds to the time period of the simulation. The color gradient, transitioning from green to red, indicates increasing sensitivity, with red areas representing periods where specific parameters exert greater influence on model outputs.

A key observation from the plot is that parameter sensitivity is dynamic, varying across different growth stages of the wheat crop. Some parameters show sustained influence throughout the simulation, while others exhibit temporary spikes in sensitivity, possibly corresponding to critical crop development phases such as germination, canopy expansion, or grain filling. Parameters associated with soil moisture, root depth, and evapotranspiration appear to have significant influence, particularly during periods of water stress.

Additionally, the incorporation of 90% confidence limits in the DYNIA plot provides insights into uncertainty trends. Narrower confidence intervals indicate periods when parameters are well-identified, suggesting that the available data contains strong information about those parameters. Conversely, wider intervals imply increased uncertainty and potential parameter interaction effects. The results suggest that sensitivity analysis using DYNIA can help refine calibration strategies by focusing on the most critical parameters at different time steps, ultimately improving model predictive performance.

The application of Aqua-MC and DYNIA to the Qazvin wheat case study provided critical insights into the model’s reliability and parameter sensitivity. The AquaCrop model was calibrated using wheat yield and meteorological data from the Qazvin synoptic station, spanning 36 years (1979–2014). A total of 3000 Monte Carlo simulations were performed on 47 crop parameters, evaluating their impact on five key output variables: soil evaporation, crop transpiration, evapotranspiration, crop biomass at maturity, and grain yield. The results showed that the 10% threshold of the Normalized Root Mean Square Error (NRMSE) was a valid criterion for defining acceptable model outputs. However, it was observed that evaporation and yield rates exhibited the highest uncertainty, while transpiration and biomass were more reliable outputs. The study highlighted that around half of the tested parameters were ineffective in influencing the model outputs, suggesting that parameter reduction could enhance the model’s efficiency.

The algorithm and codes in MATLAB are provided in Appendix 1 as a source of this study which can be used for other researchers and could be also used for other closed-source models. While our findings validate the utility of Aqua-MC for AquaCrop, it is important to acknowledge that the approach has not yet been tested on other closed-source models. Although the underlying principles of Monte Carlo simulations and automation are broadly applicable, further research is required to assess adaptability, computational performance, and compatibility with other closed-source modeling frameworks. Future studies should explore its applicability to other process-based models beyond AquaCrop, particularly in fields such as hydrology, climate modeling, and ecosystem simulations.

## Summary and outlook

The Monte Carlo method is widely used for uncertainty analysis and model behavior assessment, providing valuable probabilistic insights. However, applying this approach to closed-source models presents significant challenges compared to open-source alternatives, often discouraging researchers from conducting such analyses. The primary limitation lies in the inaccessibility of source code, which complicates the implementation of batch simulations and sensitivity analyses in closed-source frameworks.

To address this issue, we developed Aqua-MC, an automated framework designed to facilitate Monte Carlo simulations in AquaCrop, a widely used closed-source crop model. Aqua-MC integrates probabilistic parameter selection, iterative model execution, and uncertainty quantification within a structured workflow. Using the SAFE toolbox in MATLAB, Aqua-MC streamlines sensitivity analysis and likelihood evaluation, reducing the complexity of running AquaCrop simulations manually. By enabling efficient testing of thousands of parameter sets, it minimizes user intervention while ensuring robust uncertainty assessments and dynamic model characterization.

As a practical demonstration, we applied Aqua-MC and the DYNIA method to analyze wheat yield in Qazvin, a region with specific climate conditions and water-limited agriculture. The algorithm allowed us to conduct a comprehensive uncertainty analysis, identifying key parameters influencing wheat growth under varying environmental conditions. The results highlighted the dynamic nature of parameter sensitivity, reinforcing the necessity of time-dependent sensitivity analysis in crop modeling.

Despite its advantages, Aqua-MC has certain limitations. The algorithm relies on Generalized Likelihood Uncertainty Estimation (GLUE), which assumes that all model realizations meeting a given likelihood threshold are equally probable^50,51^. This assumption may lead to overestimated uncertainty bounds and less precise parameter estimation. Additionally, the computational demand associated with running thousands of simulations makes it necessary to optimize processing efficiency.

To enhance the efficiency and applicability of Aqua-MC, future studies can consider several approaches, such as (1). Optimizing computational workflows through parallel processing or cloud-based execution to reduce runtime; (2). Integrating machine learning techniques to improve parameter selection and predictive accuracy; (3). Expanding applications to multi-crop studies and diverse agro-climatic regions to test model robustness; and (4). Incorporating remote sensing data for real-time calibration, particularly in data-scarce regions.

By addressing these challenges, Aqua-MC can further improve uncertainty analysis and model calibration, making closed-source crop models more accessible and applicable for large-scale agricultural research.

## Electronic supplementary material

Below is the link to the electronic supplementary material.

Supplementary Material 1

## Data Availability

All data are available within the article and supplementary file.
