# Supplementary material for: Aqua-MC as a simple open access code for uncountable runs of AquaCrop
Source: Sci Rep. 2025 Jul 10;15:24975. doi: 10.1038/s41598-025-08995-z (PMC12246458; doi:10.1038/s41598-025-08995-z)
Supplement: Supplementary file 1 — Supplementary Material 1 [file 41598_2025_8995_MOESM1_ESM.docx]

The four main functions of Aqua-MC that was written by MATLAB 2013 are in below.

**1.**

**function Sampling_Treatments_AAT**

**%% this function do sampling random between maximum and minimum of inputs values** **all at a time (AAT)**

**N=200000;**

**addpath('GSA thesis')**

**%% Sampeling from SAFE toolbox (pianosi et. all 2015) by AAT_sampling(SampStrategy,1,DistrFun,DistrPar,N)**

**addpath(genpath('SAFE'))**

**DistrFun ='unif';**

**SampStrategy ='lhs';**

**%%**

**wheat_statics=[NaN;5;1;2;1;0;0;0;26;2151;0.150;0.60;0;0.50;0;0.650;2.50;0;...**

**0.850;0;50;2.550;0.790;1.330;-0.30;3.520;5;35;14;2;15;0;1.10;0.150;0.30;1.20;...**

**15;0.0430;0.0130;50;1.50;1.50;3750000;0.03910;-9;-9;-9;1;0.062520;12;71;191;236;...**

**142;34;0;100;83;15;100;51;50;5;10;7;15;134;419;1335;2151;725;428;0.0066550;0.0034480;1204]; % first empty cropfile values for wheat**

**for m=2:N;wheat_statics(:,m)=wheat_statics(:,1);end**

**u=[7;8;9;11;12;13;14;15;16;17;19;20;27;28;29;30;31;33;34;35;36;37;...**

**38;39;40;41;42;43;44;48;49;50;51;52;53;54;55;58;59;60;61;62;63;64;65;66]; % u: line number of changable parameters in cropfile**

**u(31:38)=[];**

**u(39:47)=67:75;**

**ef=[7 10 18 20 21 27 28 30 31 37 40 43 56 57 60 61 62 63 66 67 68 69 70 71 72 75];% ef: parameters that cant accept float number**

**wheat_range_of_inputs=[0,2;0,10;24,35;0.110,0.350;0.40,0.990;0,10;0.20,0.90;0,5;0.40,0.990;0,5;0,2;0,10;0,10;20,50;8,20;...**

**0,10;10,26;0.70,1.50;0,0.50;0.20,0.40;0,2.10;8,21;0,0.0650;0,0.030;35,65;1,2;1,2;52630,105000;0,0.30;0.60,1.20;...**

**10,50;70,130;35,65;33,63;0,10;2,14;0,9;10,20;20,170;1120,2200;1100,2400;1000,2400;500,1500;100,900;0.0010,...**

**0.030;0.0010,0.03;500,1300];% range of crop parameters**

**xmin=nan(75,1);xmin(u)=wheat_range_of_inputs(:,1);**

**xmax=nan(75,1);xmax(u)=wheat_range_of_inputs(:,2);**

**st=nan(75,N);**

**st(2,:)=5.0;st(3,:)=1;st(4,:)=2;st(5,:)=1;st(6,:)=0;st(10,:)=2400;st(18,:)=5;st(21,:)=50;st(22,:)=25;st(23,:)=25;st(24,:)=25;st(25,:)=25;st(26,:)=25;st(32,:)=0;% unchangeble variables**

**st(45,:)=-9 ;st(46,:)=-9 ;st(47,:)=-9 ;st(56,:)=1;st(57,:)=100;**

**st(2,:)=wheat_statics(2,1);st(3,:)=wheat_statics(3,1);st(4,:)=wheat_statics(4,1);st(5,:)=wheat_statics(5,1);st(6,:)=wheat_statics(6,1);st(10,:)=wheat_statics(10,1);st(18,:)=wheat_statics(18,1);**

**st(21,:)=wheat_statics(21,1);st(22,:)=wheat_statics(22,1);st(23,:)=wheat_statics(23,1);st(24,:)=wheat_statics(24,1)**

**st(25,:)=wheat_statics(25,1);st(26,:)=wheat_statics(26,1);st(32,:)=wheat_statics(32,1);% unchangeble variables**

**st(45,:)=wheat_statics(45,1) ;st(46,:)=wheat_statics(46,1) ;st(47,:)=wheat_statics(47,1) ;st(56,:)=wheat_statics(56,1);st(57,:)=wheat_statics(57,1);**

**st(49:55,:)=wheat_statics(49:55,:);st(58,:)=wheat_statics(58,:);**

**%%**

**for h=1:N**

**s=0;g=1;**

**for k=2:75**

**if isnan (st(k,h))**

**if k~=ef**

**DistrPar = [xmin(k) xmax(k)]**

**st(k,:) = AAT_sampling(SampStrategy,1,DistrFun,DistrPar,N) %acceptable float inputs**

**else**

**st(k,:)=randi([floor(xmin(k)),ceil(xmax(k))],1,N);%%% dont acceptable float inputs**

**end**

**end**

**end**

**end**

**end**

**st=st(2:75,:);**

**save sampled_random_cropfile.mat st %**

**2.**

**function Write_Treatments**

**sample=load('sampled_random_cropfile','-mat');%**

**N=200000;%**

**for n=1:N**

**%% write treatment notes ascii**

**fid=fopen('C:\Users\Documents\MATLAB\Empty Model\Empty.CRO');**

**A=textscan(fid,'%s','delimiter','\n','CollectOutput',0);**

**A=char(A{1,1});**

**c=A;**

**a=c(1:end,1:end);**

**close all**

**%% create new folder for n'th treatment and duplicate the empty model files on it**

**addpath(['D:\Users\Treatments' '\' num2str(n)]);%**

**mkdir(['D:\Users\Treatments' '\' num2str(n)]);%**

**copyfile(['C:\Users\Documents\Empty Model'],['D:\Users\Treatments' '\' num2str(n) '\' 'LIST'],'f');%**

**copyfile('C:\Users\Documents\ACsaV60Nr17042017',['D:\Users\Treatments' '\' num2str(n) ],'f'); %**

**%% write sample crop data on n'th treatment folder**

**st=num2str(sample(:,n));**

**for y=1:74;fs(y,1)='^';end**

**as=char();**

**as(2:75,:)=strcat(st,fs,a(2:end,:));**

**as(1,1:112)=a(1,:);**

**siz=size(as);**

**f=fopen(['D:\Users\Treatments' '\' num2str(n) '\' 'LIST' '\' 'WheatGDD1.CRO'],'w');**

**format={'%s \r\n','%0.10f %s \r\n' };**

**for m=1:siz(1)**

**ds=strfind(as(m,:),'^');**

**as(m,ds)=' ';**

**fprintf(f,format{1},as(m,:));**

**end**

**fclose all**

**end**

**3.**

**function Run_All_exe**

**N=20000;**

**for n=1:N**

**open (['D:\Users\Treatments' '\' num2str(n) '\' 'ACsaV60.exe']); % location of generated treatments files**

**pause(6) %approximately delay(s) for run one by one and CPU rest, concerns to hardware systems**

**end**

**4.**

**function Output_Read_and_Conclusion**

**N=200000;% number of random treatments**

**observed_output=['C:\Users\Qazvin_Wheat\ACsaV60Nr17042017\OUTP\QazvinWheatPRMseason.OUT']; % location of field measured data**

**for r=1:N**

**predicted_output=['D:\Users\Treatments' '\' num2str(r) '\' 'OUTP' '\' 'QazvinWheatPRMseason.OUT']; % location of simulated outputs**

**%% Read actual field measured data that called "Observed output"**

**fid=fopen(observed_output);**

**f='%9s%9s%9s%9s%9s%9s%9s%9s%9s%9s%9s%9s%9s%9s%9s%9s%9s%9s%10s%10s%10s%10s%9s%9s%9s%9s%9s%9s%9s%10s%9s%9s%9s%9s%9s%9s%9s%s%[^\n\r]';**

**C=textscan(fid, '%[^\n\r]', 3, 'ReturnOnError', false);**

**D= textscan(fid, f, 'Delimiter', '', 'WhiteSpace', '', 'ReturnOnError', false);**

**Irri_O=D{9}(1:end);Irri_O=cell2mat(Irri_O);Irri_O=str2num(Irri_O);**

**E_O=D{14}(1:end);E_O=cell2mat(E_O);E_O=str2num(E_O);**

**Tr_O=D{16}(1:end);Tr_O=cell2mat(Tr_O);Tr_O=str2num(Tr_O);**

**Biomass_O=D{30}(1:end);Biomass_O=cell2mat(Biomass_O);Biomass_O=str2num(Biomass_O);**

**yield_O=D{33}(1:end);yield_O=cell2mat(yield_O);yield_O=str2num(yield_O);yield_O(find(yield_O==-9))=0 ;**

**%%%%%%%%%%%%%%%%%%%%%%%%%%%%%%%**

**Irri_O=Irri_O(find(yield_O~=0));**

**E_O=E_O(find(yield_O~=0));**

**Tr_O=Tr_O(find(yield_O~=0));**

**ET_O = E_O + Tr_O ;**

**Biomass_O=Biomass_O(find(yield_O~=0));**

**yield_O=yield_O(find(yield_O~=0));**

**%%%%%%%%%%%%%%%%%%%%%%%%%%%%%%%%**

**mu_Irri_O=sum(Irri_O)/length(Irri_O); %average = mu**

**mu_E_O=sum(E_O)/length(E_O);**

**mu_Tr_O=sum(Tr_O)/length(Tr_O);**

**mu_ET_O=sum(ET_O)/length(ET_O);**

**mu_Biomass_O=sum(Biomass_O)/length(Biomass_O);**

**mu_yield_O=sum(yield_O)/length(yield_O);**

**%% Read created treatment outputs (Biomass, Evapotranspiration, Yield and ...) from 1 to 200000**

**fid=fopen(predicted_output);**

**if fid==-1;continue,end**

**f='%9s%9s%9s%9s%9s%9s%9s%9s%9s%9s%9s%9s%9s%9s%9s%9s%9s%9s%10s%10s%10s%10s%9s%9s%9s%9s%9s%9s%9s%10s%9s%9s%9s%9s%9s%9s%9s%s%[^\n\r]';**

**C=textscan(fid, '%[^\n\r]', 3, 'ReturnOnError', false);**

**D= textscan(fid, f, 'Delimiter', '', 'WhiteSpace', '', 'ReturnOnError', false);**

**if length(D{32}(1:end))<210;continue;end**

**HI=D{32}(1:end);**

**HI=cell2mat(HI);**

**HI=str2num(HI); %%%%just for identify totals matrix**

**g=size(HI)**

**if g(1)<210;continue;end**

**Irri_P=D{9}(1:end);Irri_P=cell2mat(Irri_P);Irri_P=str2num(Irri_P);**

**E_P=D{14}(1:end);E_P=cell2mat(E_P);E_P=str2num(E_P);**

**Tr_P=D{16}(1:end);Tr_P=cell2mat(Tr_P);Tr_P=str2num(Tr_P);**

**Biomass_P=D{30}(1:end);Biomass_P=cell2mat(Biomass_P);Biomass_P=str2num(Biomass_P);**

**yield_P=D{33}(1:end); s1=size(yield_P);yield_P=cell2mat(yield_P);yield_P=str2num(yield_P);%yield_P(find(yield_P==-9))=0 ;**

**%%%%%%%%%%%%%%%%%%%%%%%%%%%%%%%**

**Irri_P=Irri_P(find(HI~=-9));**

**E_P=E_P(find(HI~=-9));**

**Tr_P=Tr_P(find(HI~=-9));**

**ET_P = E_P + Tr_P ;**

**Biomass_P=Biomass_P(find(HI~=-9));**

**yield_P=yield_P(find(HI~=-9));**

**if length(yield_P)==length(yield_O)**

**%% Estimate statistics and mathematical coefficients by simulated data and actual field data**

**%% Estimate nash-suttclif coefficient**

**NS_Irri(r,1)=1-sum((Irri_P - Irri_O).^2)/sum((Irri_O - mu_Irri_O).^2);**

**NS_E(r,1)=1-sum((E_P - E_O).^2)/sum((E_O - mu_E_O).^2);**

**NS_Tr(r,1)=1-sum((Tr_P - Tr_O).^2)/sum((Tr_O - mu_Tr_O).^2);**

**NS_ET(r,1)=1-sum((ET_P - ET_O).^2)/sum((ET_O - mu_ET_O).^2);**

**NS_Biomass(r,1)=1-sum((Biomass_P - Biomass_O).^2)/sum((Biomass_O - mu_Biomass_O).^2);**

**NS_yield(r,1)=1-sum((yield_P - yield_O).^2)/sum((yield_O - mu_yield_O).^2);**

**%% Estimate NRMSE coefficient**

**NRMSE_Irri(r,1)=((((sum((Irri_P - Irri_O).^2))/length(Irri_P))^.5)/mu_Irri_O)*100;**

**NRMSE_E(r,1)=((((sum((E_P - E_O).^2))/length(E_P))^.5)/mu_E_O)*100;**

**NRMSE_Tr(r,1)=((((sum((Tr_P - Tr_O).^2))/length(Tr_P))^.5)/mu_Tr_O)*100;**

**NRMSE_ET(r,1)=((((sum((ET_P - ET_O).^2))/length(ET_P))^.5)/mu_ET_O)*100;**

**NRMSE_Biomass(r,1)=((((sum((Biomass_P - Biomass_O).^2))/length(Biomass_P))^.5)/mu_Biomass_O)*100;**

**NRMSE_yield(r,1)=((((sum((yield_P - yield_O).^2))/length(yield_P))^.5)/mu_yield_O)*100;**

**%% Estimate Willmott 1982 agreement coefficient**

**willmott_Irri(r,1)=1-(sum((Irri_P - Irri_O).^2))/sum((abs(Irri_P - mu_Irri_O)+abs(Irri_O - mu_Irri_O)).^2);**

**willmott_E(r,1)=1-(sum((E_P - E_O).^2))/sum((abs(E_P - mu_E_O)+abs(E_O - mu_E_O)).^2);**

**willmott_Tr(r,1)=1-(sum((Tr_P - Tr_O).^2))/sum((abs(Tr_P - mu_Tr_O)+abs(Tr_O - mu_Tr_O)).^2);**

**willmott_ET(r,1)=1-(sum((ET_P - ET_O).^2))/sum((abs(ET_P - mu_ET_O)+abs(ET_O - mu_ET_O)).^2);**

**willmott_Biomass(r,1)=1-(sum((Biomass_P - Biomass_O).^2))/sum((abs(Biomass_P - mu_Biomass_O)+abs(Biomass_O - mu_Biomass_O)).^2);**

**willmott_yield(r,1)=1-(sum((yield_P - yield_O).^2))/sum((abs(yield_P - mu_yield_O)+abs(yield_O - mu_yield_O)).^2);**

**Resault(r,:)=[NS_Irri(r,1),NS_E(r,1),NS_Tr(r,1),NS_ET(r,1),NS_Biomass(r,1),NS_yield(r,1),...**

**NRMSE_Irri(r,1),NRMSE_E(r,1),NRMSE_Tr(r,1),NRMSE_ET(r,1),NRMSE_Biomass(r,1),NRMSE_yield(r,1),...**

**willmott_Irri(r,1),willmott_E(r,1),willmott_Tr(r,1),willmott_ET(r,1),willmott_Biomass(r,1),willmott_yield(r,1),...**

**RMSE_Irri(r,1),RMSE_E(r,1),RMSE_Tr(r,1),RMSE_ET(r,1),RMSE_Biomass(r,1),RMSE_yield(r,1)];**

**%% Save resualts on an xls file**

**range=['C' num2str(r) ':' 'Z' num2str(r)];**

**sheet='1';**

**xlswrite('Qazvin_Wheat_Resaults.xls',Resault(r,:),sheet,range);**

**end**

**fclose all**

**end**

**end**

*An important point is the project files (.PRM) that duplicated from empty model must including correct addresses of themselves input files. If the files addresses are different from the origin, by adding below code to the function of write treatments, this problem will be solved:

**function Edit_PRM**

**N=200000;% number of treatments**

**length_PRM=1423;** **% number of .PRM script lines**

**for n=1:N**

**fide=fopen(['D:\Users\Treatments' '\' num2str(n) '\' 'LIST' ‘\’ 'QazvinWheat.PRM']);**

**M=textscan(fide,' %s','delimiter','\n','CollectOutput',0);**

**for h=60:40:length_PRM;**

**s=0;**

**for x=h-30:3:h**

**s=s+1;**

**if (s~=12)**

**M{1,1}{x,:}=['C:\Users\Treatments '\' num2str(n) '\' 'LIST' '\'];**

**else**

**M{1,1}{x+3,:}=['C:\Users\Treatments '\' num2str(n) '\' 'LIST' '\'];**

**end**

**end**

**end**

**fi=fopen(['C:\Users\Treatments '\' num2str(n) '\' 'LIST' '\' 'QazvinWheat.PRM'],'w');**

**fprintf(fi,' %s \r\n',M{1,1}{:,:});**

**end**

**end**

*Appendix 2.*

The name of crop parameters and sensitivity graphs are attached in below.

**Table 1. Name of crop parameters that able to vary in this study (* Pararmeters must be integer number that AquaCrop can’t accept float number for them) (Adabi et al., 2023).**

| **Name of Crop Parameters** |  |
| --- | --- |
| **Soil water depletion factors (p) are adjusted by Eto** | **X1 *** |
| **Base temperature (°C) below which crop development does not progress** | **X2** |
| **Upper temperature (°C) above which crop development no longer increases with an increase in temperature** | **X3** |
| **Soil water depletion factor for canopy expansion (p-exp) - Upper threshold** | **X4** |
| **Soil water depletion factor for canopy expansion (p-exp) - Lower threshold** | **X5** |
| **Shape factor for water stress coefficient for canopy expansion (0.0 = straight line)** | **X6** |
| **Soil water depletion fraction for stomatal control (p - sto) - Upper threshold** | **X7** |
| **Shape factor for water stress coefficient for stomatal control (0.0 = straight line)** | **X8** |
| **Soil water depletion factor for canopy senescence (p - sen) - Upper threshold** | **X9** |
| **Shape factor for water stress coefficient for canopy senescence (0.0 = straight line)** | **X10** |
| **Soil water depletion factor for pollination (p - pol) - Upper threshold** | **X11** |
| **Vol% for Anaerobiotic point (* (SAT - [vol%]) at which deficient aeration occurs *)** | **X12 *** |
| **Minimum air temperature below which pollination starts to fail (cold stress) (°C)** | **X13 *** |
| **Maximum air temperature above which pollination starts to fail (heat stress) (°C)** | **X14 *** |
| **Minimum growing degrees required for full biomass production (°C - day)** | **X15 *** |
| **Electrical Conductivity of soil saturation extract at which crop starts to be affected by soil salinity (dS/m)** | **X16 *** |
| **Electrical Conductivity of soil saturation extract at which crop can no longer grow (dS/m)** | **X17 *** |
| **Crop coefficient when canopy is complete but prior to senescence (KcTr,x)** | **X18** |
| **Decline of crop coefficient (%/day) as a result of ageing, nitrogen deficiency, etc.** | **X19** |
| **Minimum effective rooting depth (m)** | **X20** |
| **Maximum effective rooting depth (m)** | **X21** |
| **Shape factor describing root zone expansion** | **X22 *** |
| **Maximum root water extraction (m3water/m3soil.day) in top quarter of root zone** | **X23** |
| **Maximum root water extraction (m3water/m3soil.day) in bottom quarter of root zone** | **X24** |
| **Effect of canopy cover in reducing soil evaporation in late season stage** | **X25 *** |
| **Soil surface covered by an individual seedling at 90 % emergence (cm2)** | **X26** |
| **Canopy size of individual plant (re-growth) at 1st day (cm2)** | **X27** |
| **Number of plants per hectare** | **X28 *** |
| **Canopy growth coefficient (CGC): Increase in canopy cover (fraction soil cover per day)** | **X29** |
| **Maximum canopy cover (CCx) in fraction soil cover** | **X30** |
| **Water Productivity normalized for ETo and CO2 (WP*) (gram/m2)** | **X31** |
| **Water Productivity normalized for ETo and CO2 during yield formation (as % WP*)** | **X32 *** |
| **Crop performance under elevated atmospheric CO2 concentration (%)** | **X33 *** |
| **Reference Harvest Index (HIo) (%)** | **X34 *** |
| **Possible increase (%) of HI due to water stress before flowering** | **X35 *** |
| **Coefficient describing positive impact on HI of restricted vegetative growth during yield formation** | **X36** |
| **Coefficient describing negative impact on HI of stomatal closure during yield formation** | **X37** |
| **Allowable maximum increase (%) of specified HI** | **X38 *** |
| **GDDays: from sowing to emergence** | **X39 *** |
| **GDDays: from sowing to maximum rooting depth** | **X40 *** |
| **GDDays: from sowing to start senescence** | **X41 *** |
| **GDDays: from sowing to maturity (length of crop cycle)** | **X42 *** |
| **GDDays: from sowing to flowering** | **X43 *** |
| **Length of the flowering stage (growing degree days)** | **X44 *** |
| **CGC for GGDays: Increase in canopy cover (in fraction soil cover per growing-degree day)** | **X45** |
| **CDC for GGDays: Decrease in canopy cover (in fraction per growing-degree day)** | **X46** |
| **GDDays: building-up of Harvest Index during yield formation** | **X47 *** |

*must be integer
